# Feasibility and Acceptability of Remotely Accessed Compensatory Cognitive Training for Japanese People With Schizophrenia: Pilot Study

**DOI:** 10.2196/70916

**Published:** 2025-08-12

**Authors:** Ritsuko Aijo, Mie Matsui

**Affiliations:** 1 Department of Neuroscience, Graduate School of Medical Sciences, Kanazawa University, Kanazawa, Japan; 2 Department of Nursing, Faculty of Health Sciences, Komatsu University, He 14-1 Mukaimotoorimachi Suehiro Campus, Komatsu, Ishikawa 923-0961, Komatsu, Japan, +81 761-48-3194; 3 Laboratory of Clinical Cognitive Neuroscience, Institute of Liberal Arts and Science, Kanazawa University, Kanazawa, Japan

**Keywords:** schizophrenia, remote, CCT, compensatory cognitive training, case study

## Abstract

**Background:**

Compensatory cognitive training (CCT) is an evidence-based treatment for improving cognitive function in patients with schizophrenia. However, the need for patients to commute to treatment sites hinders its widespread use. Using a remote device to conduct CCT could improve its accessibility, making it easier for participants to adjust their schedules and reducing their burden.

**Objective:**

The objective of this study was to (1) investigate the creation and participant acceptability of CCT using a remote compensatory cognitive training (r-CCT) device, (2) determine the feasibility of implementing the developed intervention, and (3) collect preliminary data for future studies of the effectiveness of r-CCT in Japan.

**Methods:**

To reduce participant movement during training, CCT was conducted remotely in real time, using borrowed iPads. The training was conducted in a group format through video conferencing once a week for 2 h, for a total of 12 sessions. In total, 4 patients with schizophrenia who underwent r-CCT were recruited to determine participation or dropout rates across 12 training sessions. In addition, their diagnostic assessment (the Scale of Positive Symptoms and the Scale of Negative Symptoms), cognitive function (eg, the Japanese version of the Trail Making Test Part A [TMT-A] and Trail Making Test Part B [TMT-B], digit span, and digit symbol), social functioning (Social Functioning Scale Japanese version [SFS-J]), and quality of life (Japanese Schizophrenia Quality of Life Scale [JSQLS]) were assessed before, immediately after, and 3 months after implementation.

**Results:**

The average participation rate of the 3 participants (a male in his 30s was excluded) was high at 92%. Immediately after the r-CCT, positive trends were observed in cognitive function―excluding prospective memory. For example, the TMT-A scores improved for all 3 participants: Participant A (from 58 s to 56 s), Participant B (from 52 s to 49 s), and Participant C (from 65 s to 49 s). The Japanese Verbal Learning Test (JVLT) immediate scores also increased: Participant A (from 16 to 19), Participant B (from 13 to 14), and Participant C (from 14 to 21). Functional outcomes, assessed using the SFS-J, showed limited improvement immediately postintervention but tended to return to or fall below preintervention levels at the 3-month follow-up. Quality of life (QOL) scores, measured using the JSQLS, remained relatively stable or improved immediately following the r-CCT and at the 3-month follow-up.

**Conclusions:**

Despite this study’s small number of participants and lack of randomization, it suggests that the accessibility and implementation potential of r-CCT may be high. The ability to participate in training from any location could be expected to increase participation rates or reduce dropout rates. In the future, the authors will develop the implementation method further and increase the sample size to demonstrate the training’s effectiveness.

## Introduction

Schizophrenia is a chronic mental disorder that often negatively affects the daily life of individuals living with the condition, including their community and social life, due to positive and negative symptoms as well as cognitive dysfunction [1]. The main treatment for schizophrenia is pharmacotherapy, with antipsychotics primarily improving positive symptoms but affecting negative symptoms and cognitive dysfunction less [2]. Because negative symptoms and cognitive dysfunction contribute to a lower quality of life (QOL) and lifelong disability in patients with schizophrenia [3,4], emphasis is placed on improving their cognitive function [5].

Cognitive remediation therapy (CRT) is an evidence-based therapy used to improve cognition in patients with schizophrenia. The therapeutic goal is to positively impact their ability to function and live in the community through improved cognitive function [6,7]. At the 2010 International Schizophrenia Research Conference in Florence, Italy, CRT for schizophrenia was defined as “a behavioral training-based intervention aimed at improving cognitive processes (attention, memory, executive functioning, social cognition, or metacognition) with persistence and generalization [7].” The most recent meta-analysis of cognitive remediation found small-to-moderate effects in cognitive tests as well as in psychosocial function and psychiatric symptom severity [7,8]. Current treatment guidelines suggest long-term treatment with antipsychotic medication in conjunction with psychological interventions for people with psychosis [1].

Patients with schizophrenia may be hesitant to approach traditional mental health treatment settings due to stigma, which may prevent them from engaging in help-seeking behaviors [9]. Typically, clinicians run CRT sessions at least twice a week to ensure that participants have adequate practice with the cognitive exercises, which are usually provided over the internet [6]. However, multiple in-person sessions each week can pose transportation and scheduling burdens on participants, which could limit their ability to participate [10]. A study reported that reducing the need to travel to once a week may encourage more people to participate in such training [6]. In sum, while CRT exhibits efficacy, it imposes burdens on participants. These factors lead to lower CRT participation rates and higher dropout rates, which in turn significantly affect CRT’s penetration rate [11]. Therefore, it is first necessary to develop training that patients with schizophrenia can receive without the need to move to the greatest extent possible, as well as to establish the acceptability of the training for participants and the feasibility of implementing it.

Accordingly, this study aimed to evaluate the acceptability of CRT training by examining participation and dropout rates. Furthermore, it aimed to examine whether compensatory cognitive training (CCT), a type of CRT, improves patients’ cognitive function, daily life functioning, and QOL as a result of its implementation at a remote site using quantitative methods. Furthermore, the study aimed to examine the feasibility of remote compensatory cognitive training (r-CCT) implementation in Japan. However, if patients with schizophrenia lack experience with computers, they could experience frustration in learning if a clinician is not present for support and guidance [10]. Thus, to support participants’ acceptance and continuation of the r-CCT, only the therapist participated remotely while the participants attended sessions together at a location where an assistant was available to provide technical and other support. The meeting locations for the r-CCT sessions were places that the participants commuted to on a daily basis and did not involve longer travel. To collect preliminary data for a future study of the effectiveness of r-CCT in Japan, r-CCT was administered to 4 patients with schizophrenia in this study. The remote approach to CCT piloted in this study is, to our knowledge, the first to be evaluated in Japan. Furthermore, a quantitative approach was used to examine the training’s acceptability to the patients as well as the feasibility of its implementation.

## Methods

### Recruitment

The 4 participants were recruited from different social welfare facilities in Japan, specifically from 4 facilities in proximity to each other. To avoid selection bias in participant recruitment, facility administrators—rather than researchers—approached the users themselves. While the patients belonged to the same social welfare facility, 3 of them worked at the same location and 1 worked somewhere else. The r-CCT was conducted at the location where the 3 participants normally worked; therefore, only 1 participant was required to move to participate in the study. The inclusion criteria were as follows: (1) aged 18 years or older, a diagnosis of schizophrenia according to the *Diagnostic and Statistical Manual of Mental Disorders, Fifth Edition* or the *International Classification of Diseases 10th Revision*, fluency in Japanese, and stable enough to participate in group treatment. The exclusion criteria were as follows: head trauma, cerebrovascular and neurodegenerative disorders, diagnosis of alcohol and drug dependence, and a premorbid estimated IQ of 70 on the Japanese version of the Adult Reading Test [12].

Data were collected between February and September 2022. The 4 participants (3 men and 1 woman) had a mean age of 55.8 years, mean age of onset of illness of 30 years, mean duration of illness of 27 years, mean education of 12.8 years, estimated IQ before illness onset of 101.3, and mean daily dose of antipsychotic medication (chlorpromazine equivalent) of 1163.8 mg. All of them worked at a job of support B-type; however, their job descriptions and working hours differed. Of the 4 participants, 1 used a cellphone and 1 used a smartphone, but none had ever used an iPad or a computer. Table 1 presents detailed information on each participant. Initially, we planned to recruit a larger number of participants; however, due to a low number of applicants at the target facilities and practical constraints imposed by the COVID-19 pandemic, only 4 participants ultimately participated. Among them, Participant D exhibited pronounced positive symptoms of schizophrenia at the start of the intervention and, at their own request, was excluded from most of the program, completing only 2 training sessions. Consequently, they were considered to have withdrawn from the study and were excluded from the analyses of cognitive function, functional outcomes, and QOL.

**Table 1.** Sample characteristics before compensatory cognitive training using a remote compensatory cognitive training (r-CCT) device intervention for individuals diagnosed with schizophrenia who participated in this pilot study. Participants were recruited from social welfare facilities in Japan between February and September 2022.

Participant	Sex	Age (years)	Age at onset (years)	Illness duration (years)	Education (years)	Occupation	Use of internet-based equipment	Estimate of premorbid IQ	Antipsychotic dose (mg/day)^a^
A	Male	68	28	40	12	During the day, they go to a job support B-type, in which they work for approximately 2 h a day, doing coffee shop work and cooking.	No previous experience with cell phones, smartphones, iPads, and personal computers.	104	1600
B	Male	54	40	15	12	During the day, they attend a job support type B, in which they mainly work for 1‐2 h a day bagging electronic components.	No previous experience with cell phones, smartphones, iPads, and personal computers.	104	600
C	Female	64	28	36	15	During the day, they attend a job support B-type and do work commissioned by the facility for approximately 2 h a day. Sometimes, they exhibit their crocheted works.	Have used and still use cell phones; no previous experience with smart phones, iPads, and computers.	96	1200
D	Male	37	22	15	12	During the day, they go to a job support B-type, in which they inspect goods, process food, and pack bags for approximately 3 h a day. However, even within those 3 h, frequent breaks are required because of the illness.	Have used and still use cell phones and smart phones; no previous experience with iPad and personal computers	101	1255

a Antipsychotic medication dose is chlorpromazine equivalent (mg/day).

### Procedures

The r-CCT’s acceptability was assessed by examining the 4 patients’ participation and dropout rates across the 12 training sessions. In addition, the feasibility of implementing r-CCT in Japan was evaluated by assessing the participants’ cognitive function, social functioning, and QOL at baseline, 3 months (ie, postintervention), and 6 months. The goal here was to determine whether they exhibited trends similar to those of patients who receive face-to-face CCT.

### CCT Intervention and Remote Methods

CCT is a type of cognitive remediation training developed that targets the following 4 cognitive domains: prospective memory, attention, learning and memory, and executive functioning [13]. The r-CCT used in this study is not a newly developed method of intervention but is based on an existing manualized CCT training developed by Twamley et al [13]. The Japanese version of CCT had already been translated and culturally adapted by researchers independent of the research team. In this study, the basic treatment structure and session content were maintained; however, the format was modified to accommodate remote delivery. The changes include sharing Microsoft PowerPoint files through the internet-based meeting platform (Microsoft Teams), writing session content on the slides in real time, and using audio and video equipment. The compensatory strategies trained are both internal (eg, learning and recollection of information using acronyms or visual imagery) and external (eg, learning and recollection of information by writing it down somewhere it will be seen). These strategies lead to a wide range of improvements, ranging from those in cognitive function to social functioning to QOL [13]. The CCT intervention is a face-to-face, manual training program that comprises 4 to 6 participants and several therapists, who meet once a week for 2 hours for a total of 12 sessions [13]. Each session uses a workbook and an interactive format that allows for interactivity between participants and therapists as well as among participants [13]. In this study, a workbook originally translated into Japanese was used, and the approach of manual group therapy for 2 hours per week for 12 weeks was followed [14]. During the training, participants learn many strategies, practice their use in CCT, and habitually use them in the real world—even after the CCT is over [13]. A simple homework assignment (eg, carry a calendar with you every day) is given at the end of each session to encourage participants to continue using the strategies [13].

**Figure 1.**
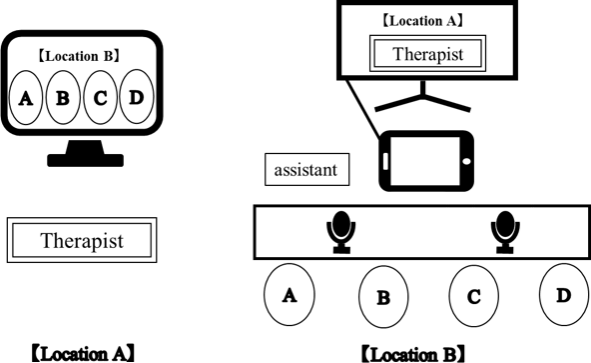
Training scene of remote compensatory cognitive training (r-CCT) for people with schizophrenia conducted at a social welfare facility in Japan. The intervention was conducted from February to September 2022.

This paper is the first report concerning CCT performed remotely. To emphasize technical assistance, the 4 participants were asked to undergo r-CCT at the same location, assisted by 1 or 2 assistants. The remote approach involved placing 1 Apple iPad Pro in a room with the participants and 1 assistant so that the therapist could communicate with them internet-based from another room. A Microsoft Teams was used for internet-based interaction. For video, iPad images were projected onto a TV screen so that all participants could view them under the same conditions. For audio, 2 speakerphones (eMeet Office Core) were used to easily pick up the voices of multiple participants. At the first training session, workbooks and remote equipment were given to the participants and assistants (see Figure 1).

The assistant was asked to connect 1 iPad and 2 eMeet Office Core devices, project the iPad video, and set up participation in a Microsoft Teams meeting. In this study design, participants were not expected to operate iPads; therefore, all remote device operations were performed by assistants. However, rather than increasing the number of staff relative to that required for face-to-face training, we devised a method to conduct remote training using the same number of staff. The therapist and assistant tested the connection once at a time and date that differed from the r-CCT. The assistants were instructed to speak only to participants who experienced problems during the r-CCT, with the exception of the provision of technical support. To examine the remote method of delivery, a preliminary survey was conducted with 4 healthy people in advance. Through these preliminary interviews, the implementation of the existing CCT was confirmed to be possible through adjusting the volume and screen of the remote devices; then, the main study could begin.

### Quantitative Data Collection

The r-CCT’s acceptability was assessed by recording how many times participants were able or unable to participate during the 12 training sessions as a percentage. The feasibility of its implementation was assessed by performing the following inspections on participants before, immediately after, and 3 months after the implementation of the r-CCT. The inspections were performed by personnel trained in standardized procedures.

With regard to symptom assessment, the severity of positive symptoms was measured using the Scale of Positive Symptoms (SAPS [15]), while the severity of negative symptoms was measured using the Scale of Negative Symptoms (SANS [16]). Both the SAPS and the SANS are rated on a 6-point scale (0‐5 for each item), with higher scores indicating a greater severity of symptoms. Both scales score the severity of psychiatric symptoms based on observations and interviews with patients with schizophrenia.

In addition, a combination of existing tests was used that is thought to correspond to the 4 cognitive domains covered by CCT (ie, prospective memory, attention, learning and memory, and executive functioning). Specifically, the belongings subtests of the Rivermead Behavioral Memory Test (RBMT; Japanese version), the Trail Making Test Part A (TMT-A; Japanese version), the digit symbol and digit span subtests of the Wechsler Adult Intelligence Scale–Fourth Edition (WAIS-IV), the Japanese Verbal Learning Test (JVLT), the story recall subtest of the RBMT, and the Trail Making Test Part B (TMT-B; Japanese version). The tests were administered in a specific order each time.

First, prospective memory was assessed with the belongings subtests of the RBMT [17,18]. The belongings task involved the examiner hiding the participant’s belongings (eg, their keys and watch) under a desk or another location that was not visible to the participant at the beginning of the cognitive test; then, the participant was asked to recall the hidden belongings at the end of all cognitive tests. Thus, their memory of the need to request the belongings as well as of the hidden location was assessed. The participants received 2 points each for recalling the location and belongings without hints, and 1 point each for recalling the location and belongings with hints, for a maximum of 4 points.

Second, attention was assessed using the TMT-A [19], the digit symbol subtest of the WAIS-IV [20,21], and the digit span subtest of the WAIS-IV [20,21]. First, in the TMT-A, the participant must tie 25 numbers in order as quickly as possible without making mistakes, and the time taken to complete the task is measured. The shorter the completion time, the higher their cognitive ability. Second, in the digit symbol subtest, participants have 2 minutes to write down symbols that correspond to digits as quickly and as accurately as possible. The study used a maximum score of 19 points, with the number of correctly described signs adjusted for age. Finally, in the digit span subtest, participants must reproduce digits read aloud in the correct order. The number of digits is gradually increased and assessed by the maximum number of digits they can correctly reproduce. The maximum number of digits in this study was 9.

Third, learning and memory were assessed with the JVLT [22] and the story recall subtest of the RBMT [18]. First, the JVLT consisted of a list of 16 words divided into 4 categories. The words were presented orally, and participants were asked to recall the words for 3 trials each time the 16 words were presented. In this study, the total number of correct responses to recall over the 3 trials (ie, highest score=48) was used to evaluate the participants. Second, the story recall subtest is a task scored up to 25 points in which participants remember and replay a short story that was told orally.

Finally, executive function was assessed using the TMT-B [19]. The task involves connecting 13 numbers and 12 hiragana (1 of the 3 writing systems in Japanese), alternately in sequence (eg, 1a - 2 - i - 3u.), as quickly as possible and without making mistakes. The time taken to complete the task was measured, and the shorter the completion time, the higher the participant’s cognitive ability.

Furthermore, functional outcomes were assessed using the Japanese version of the Scale of Social Functioning (SFS-J). The SFS-J is a self-administered rating scale with 7 subscales [23]. It consists of a total of 75 items that relate to basic skills and social behaviors required for daily living, and they are assessed using subscale scores and total scores. For the question items in the subscales of withdrawal, interpersonal relationships, social participation, recreation, independence and competence, independence and performance, and employment, the participants indicated how often they perform the stated behaviors (response options: “never,” “rarely,” “sometimes,” and “frequently”) or how well they perform them (response options: “do not know how to do them,” “cannot do them,” “can do them with help,” and “can do them well enough”). Each item was scored on a 4-point scale from 0 to 3, with higher scores indicating better functioning. The total score was used in this study. QOL was assessed using the Japanese version of the Schizophrenia Quality of Life Scale (JSQLS), which is a self-administered scale that measures the QOL of patients with schizophrenia in three domains (psychosocial relationships, motivation and vitality, and symptoms and side effects). It consists of 30 items. Responses are rated on a 5-point scale from 0 to 4, with lower scores indicating a higher QOL [24].

### Statistical Analyses

This was a preliminary study using a small sample size to examine the feasibility and acceptability of remote compensatory cognitive function improvement therapy. Due to the very small sample size (n=3), statistical tests (such as calculations of significance or effect size) were not performed. Instead, we provided a descriptive report on r-CCT participation rates and changes in each participant’s measurements, ensuring confidentiality. In addition, we investigated the feasibility and potential effects of the intervention, showing trends at three points in time: before r-CCT (T1), immediately after r-CCT (T2), and 3 months post r-CCT (T3).

### Ethical Considerations

This study was approved by the Medical Ethics Committee of Kanazawa University (approval 2020‐327 [089]) and was conducted in accordance with the principles of the Declaration of Helsinki. All participants were informed of the study’s purpose and procedures in writing and orally, and written informed consent was obtained from each participant before participation. All data collected were anonymized, and personally identifiable information was removed before analysis. This study and its supplementary materials do not contain any images or information that could identify individuals. Participants did not receive any monetary compensation; however, the intervention sessions were provided for free, and technical support was offered to ensure the participants could engage in the training sessions with confidence and ease. This study is not a secondary analysis of existing data but a new study in which participants were recruited and consent was obtained. Informed consent waivers were not applied.

## Results

### Acceptability of r-CCT to Participants

Of the 12 r-CCT sessions, Participant A attended 75% (9/12) of them, Participant B and Participant C attended 100% (12/12), and Participant D attended 17% (2/12). The average participation rate of the 4 participants was 73%, while that of the 3 participants excluding Participant D was 92%. Participant D had the lowest participation rate because he, unlike the other participants, had to drive for 15 minutes to attend the training sessions. Furthermore, they exhibited strong positive symptoms from the first session and were only able to participate in the fourth and fifth sessions from start to finish. A staff member who knew Participant D stated the following: “Mr. D sometimes said it was hard for them to leave the house when their auditory hallucinations got worse.” Participant A was absent from 3 of the 12 r-CCT sessions but never forgot their training; one of their absences was due to ill health while 2 were deliberate. The reason for their deliberate absences was an unpleasant experience during the r-CCT, which was resolved through discussion with the therapist. It was caused by emotional discomfort rather than technical difficulties or physical risks. Specifically, the therapist’s comments (eg, praising other participants) may have induced feelings of jealousy and dissatisfaction, as they did not align with the participants’ expectations. This concern was raised by participants during discussions with the research team, and through these discussions, the participants were able to gain a clearer understanding of the situation, enabling them to continue with the intervention without dropping out. Participant B was able to participate in all r-CCT sessions; however, they exhibited little voluntary participation and were often asked to participate by the staff as they forgot about the training. Participant C was able to voluntarily attend all r-CCT sessions. Regarding staff support, all of the participants indicated that they did not feel inconvenienced because the staff essentially operated the internet-based equipment. Regarding their willingness to continue the training, all participants stated that it did not matter whether staff were present or not. Furthermore, Participant C, who was absent without tardiness, said the following: “At first, I was worried that I wouldn’t understand the training, but once I did it, I had a goal and then I joined in with a challenging spirit.” Few negative comments were made about the volume and pictures on the screen. Furthermore, participants stated that they “didn’t mind that it was online,” found the content “easy to understand even online,” and described the experience as “online training that was interesting and fun, not too hard.”

### Feasibility of Implementing R-CCT

Table 2 presents the results for cognitive performance, functional outcomes, and QOL. Participant D was excluded from the analysis because they fully participated in only 2 training sessions. Therefore, this study presents the results based on data from the remaining 3 participants, excluding Participant D. Furthermore, the study design was not suitable for group comparisons or statistical significance testing. Therefore, the results are presented with a focus on changes in individual data, and descriptive statistics (such as means and SDs) are not used. This approach aims to accurately reflect the specific responses and clinical changes observed in each participant. The following sections detail these results.

**Table 2.** Changes in scores for each indicator among individuals diagnosed with schizophrenia who participated in this pilot study. Data collected from social welfare facilities in Japan between February and September 2022.

	Participant A					Participant B					Participant C					Participant D				
	T1^a^	T2^b^	T3^c^	T2–T1	T3–T1	T1	T2	T3	T2–T1	T3–T1	T1	T2	T3	T2–T1	T3–T1	T1	T2	T3	T2–T1	T3–T1
Symptom assessment																				
SAPS^d^^,^^e^ positive symptoms	14	7	2	−7^f^	−12^f^	31	30	19	−1^f^	−12^f^	6	1	2	−5^f^	−4^f^	36	48	36	+12^g^	0^h^
SANS^i^^,^^e^ negative symptoms	35	40	18	+5^g^	−17^f^	18	14	37	−4^f^	+19^g^	48	39	41	−9^f^	−7^f^	24	16	13	−8^f^	−11^f^
Prospective memory																				
Belongings (total 4 points)	4	2	4	−2^g^	0^h^	2	1	2	−1^g^	0^h^	4	2	4	−2^g^	0^h^	4	4	4	0^h^	0^h^
Attention																				
TMT-A^j^^,^^e^ (s)	58	56	60	−2^f^	+2^g^	52	49	43	−4^f^	−9^f^	65	49	38	−16^f^	−27^f^	49	52	28	+3^g^	−21^f^
Digit symbol (total 19 points)	10	11	11	+1^f^	+1^f^	6	6	8	0^h^	+2^f^	9	8	10	−1^g^	1^f^	6	4	6	−2^g^	0^h^
Digit span (total 9 digits)	5	5	6	0^h^	+1^f^	7	7	6	0^h^	−1	6	5	6	−1^g^	0^h^	6	8	7	+2^f^	+1^f^
Learning and memory																				
JVLT^k^ immediate (total 48 pieces)	16	19	15	+3^f^	−1	13	14	7	+1^f^	−5^g^	14	21	23	+7^f^	+2^f^	29	24	20	−5^g^	−9^g^
Story recall (total 25 pieces)	7.0	7.5	9.5	+0.5^f^	+2.5^f^	3.0	7.5	1.0	+4.5^f^	−2.0^g^	7.5	6.5	2.5	−1.0^g^	−5.0^g^	4.0	7.0	1.0	+3.0^f^	−3.0^g^
Executive function																				
TMT-B^l^^,^^e^ (s)	127	106	116	−21^f^	−11^f^	91	103	76	+12^g^	−15^f^	192	159	116	−33^f^	−43^f^	71	54	37	−17^f^	−34^f^
Functional outcome																				
SFS-J^m^ total score	157	147	130	−10^g^	−27^g^	112	122	113	+10^f^	+1^f^	109	114	105	+5^f^	−4^g^	106	114	121	+8^f^	+15^f^
QOL^n^																				
JSQLS^o^	57	61	56	+4^g^	−1^f^	60	45	53	−15^f^	−7^f^	55	37	43	+18^f^	+12^f^	41	41	36	0^h^	+5^f^

a T1 is before remote compensatory cognitive training (r-CCT).

b T2 is immediately after r-CCT.

c T3 is 3 months after r-CCT.

d SAPS: Scale of Positive Symptoms.

e SAPS, SANS, TMT-A, TMT-B, and SQOL indicate that the lower the score, the better the condition.

f Red indicates an increase in score.

g Blue indicates a decline in score.

h Green indicates stability of scores.

i SANS: Scale of Negative Symptoms.

j TMT-A: Trail Making Test Part A.

k JVLT: Japanese Verbal Learning Test.

l TMT-B: Trail Making Test Part B.

m SFS-J: Social Functioning Scale Japanese version.

n QOL: quality of life.

o JSQLS: Japanese Schizophrenia Quality of Life Scale.

First, the specific changes in cognitive function for Participant A, Participant B, and Participant C, as shown in Table 2, are as follows. The scores from 3 time points—before the intervention (T1), after (T2), and 3 months postintervention (T3)—are compared. Prospective memory was measured using the “belongings subtests,” with Participant A scoring 4, 2, and 4; Participant B scoring 2, 1, and 2; and Participant C scoring 4, 2, and 4. All 3 participants exhibited a decline in scores immediately after the intervention; however, their scores returned to preintervention levels at the 3-month follow-up. Attention function was measured using the TMT-A, digit symbol, and digit span subtests. In the TMT-A, shorter completion times indicated higher cognitive function. Participant A’s recorded times of 58 s, 56 s, and 60 s; Participant B recorded 52 s, 49 s, and 43 s; and Participant C recorded 65 s, 49 s, and 38 s at T1, T2, and T3, respectively. Immediately after the intervention, the performance of all 3 participants improved; however, by the 3-month follow-up, Participant A’s scores had declined, whereas Participants B and C maintained their scores. The digit symbol test, which uses a 19-point scale that takes age into account, yielded the following scores. Participant A scored 10, 11, and 11; Participant B scored 6, 6, and 8; and Participant C scored 9, 8, and 10. Immediately after the intervention, Participants A and B showed improvement; however, Participant C’s performance declined. After 3 months, all 3 participants maintained their performance levels. The digit spans were 5, 5, and 6 for Participant A; 7, 7, and 6 for Participant B; and 6, 5, and 6 for Participant C. Subsequently, the performance of Participants A and B improved, while that of Participant C declined. After 3 months, the scores of Participants A and C remained stable, whereas those of Participant B declined. Learning and memory were measured using the JVLT immediate test and the story recall test. The JVLT immediate scores were 16, 19, and 15 for Participant A; 13, 14, and 7 for Participant B; and 14, 21, and 23 for Participant C, with all 3 showing improvement in their immediate task scores. After 3 months, Participant A’s and Participant B’s scores declined, while Participant C maintained their performance levels. The story recall scores were 7.0, 7.5, and 9.5 for Participant A; 3.0, 7.5, and 1.0 for Participant B; and 7.5, 6.5, and 2.5 for Participant C. In the immediate task, Participant A’s performance improved, whereas Participant B’s and Participant C’s performance levels declined. After 3 months, Participant A maintained their earlier performance levels, whereas those of Participant B and Participant C declined. Executive function was measured using the TMT-B. In the TMT-B, shorter task completion times indicate higher cognitive function. Participant A scored 127 s, 106 s, and 116 s; Participant B scored 91 s, 103 s, and 76 s; and Participant C scored 192 s, 159 s, and 116 s. Immediately after the intervention, Participants A and C showed improvement in task performance, whereas Participant B’s performance levels declined. After 3 months, all 3 maintained their task performance levels.

To summarize the trends, with the exception of prospective memory, the attention, learning and memory, and executive functions targeted by r-CCT tended to improve immediately after r-CCT. After 3 months, the trends tended to remain stable, except for learning and memory.

Next, Table 2 shows the changes in functional outcome and QOL for Participants A, B, and C before (T1), after (T2), and 3 months after (T3) the r-CCT intervention. Functional outcome measured using the SFS-J for Participant A was 157, 147, and 130; for Participant B, it was 112, 122, and 113; and for Participant C, it was 109, 114, and 105. Immediately after the intervention, Participant A showed a decrease, whereas Participants B and C showed improvements. Furthermore, 3 months after the intervention, Participants A and B showed a decline, whereas Participant C returned to their pretraining level. QOL was measured using the JSQOL, a scale for measuring QOL in schizophrenia, with lower scores indicating higher QOL. Participant A scored 57, 61, and 56; Participant B scored 60, 45, and 53; and Participant C scored 55, 37, and 43. Immediately after the intervention, Participant A’s task performance levels decreased, while those of Participant B and Participant C showed improvement. In addition, 3 months after the intervention, all 3 maintained their QOL scores. The participants’ scores on the SFS-J, which measures functional outcomes, tended to remain stable immediately after the r-CCT. However, 3 months later, the effects had returned to pretraining levels or decreased slightly. Their scores on the JSQLS, which measures QOL, were generally maintained or improved immediately after the r-CCT, and these effects continued.

## Discussion

### Principal Findings

CRT is an evidence-based practice that is increasingly used in clinical practice to treat the widespread and significant cognitive deficits that contribute to poor functional outcomes in patients with schizophrenia [10]. Recommendations for practicing CRT include ensuring that patients receive sufficient exposure to cognitive exercises to promote cognitive change [25], usually at least twice a week [10]. Nevertheless, the challenge of attending in-person sessions several times a week may limit the treatment’s scalability and patients’ access to care. Therefore, it is crucial to consider other ways to implement CRT, which include providing opportunities for participants to access the training remotely [10].

This study was a preliminary exploratory study to examine the acceptability and feasibility of CCT using remote devices in Japanese patients with schizophrenia. As this study was the first attempt to conduct CCT entirely remotely in Japan, it was intentionally conducted with a small number of participants to confirm implementation procedures, assess participants’ responses, and identify potential challenges during the introduction phase. Due to the small sample size (n=4, with 3 completing the study) and the fact that participants were recruited from a single facility, statistical analyses were not conducted and conclusions regarding efficacy or the generalizability of results could not be drawn. However, this study provided foundational insights to inform future large-scale studies. The results obtained using this method suggest that participants with fewer movement requirements may demonstrate higher participation rates and lower dropout rates.

The 3 participants (excluding Participant D) did not have to travel to attend the r-CCT sessions, which may have facilitated their participation and resulted in fewer absences. To reduce movement, the training was delivered remotely; however, the assistants were asked to be present in the same location as the participants who were determined to require technical support and assistance with the training content. However, the assistants mostly assisted with the operation of the internet-based equipment and provided little assistance with the training content. The participants could ask the internet-based therapist any questions they had without assistance. Although the telecommunication conditions were poor and communication was sometimes choppy, no negative comments were provided by the participants. In this study, remote training was implemented as an intervention method; however, it was not a completely independent format in which participants conducted training from their homes or other locations. Instead, they visited familiar facilities and received technical support from local staff while therapists conducted the intervention remotely. This partially remote format was selected to accommodate individuals who may be unfamiliar with operating digital devices. Therefore, the results of this study do not directly indicate the feasibility of a fully remote intervention. Conclusions regarding the acceptability and scalability of r-CCT based on this study are provisional, and caution is warranted when generalizing findings to other environments.

Keef et al [26] reported that increasing the enjoyment of digital training interventions may lead to greater adherence and cognitive benefits. Furthermore, Best et al [27] reported that programs designed to increase motivation and provide opportunities to practice daily living skills improve treatment retention rates. Originally, CCT was more likely to be motivational because the sessions mainly involved group work, included fun activities such as games, and were relevant to the participants’ everyday life [28]. This study was designed to enable all of the participants to receive training in the same location, as they may have been resistant to the use of remote equipment. However, if the training itself is designed to be enjoyable and motivational, training with remote devices may not be as burdensome for those who are not used to using them. This study’s findings suggest that even remote methods may be acceptable to patients with schizophrenia if they are easy to operate. Currently, based on this study, we are conducting research on the acceptability and effectiveness of a fully home-based r-CCT to investigate its applicability in a wider range of settings.

In this study, standardized scales or structured interviews were not used to evaluate participants’ acceptability of the intervention. Instead, acceptability was primarily evaluated based on participation rates, number of absences, and anecdotal feedback. This represents one of the study’s limitations. However, as this was the first study to implement CCT remotely, its primary aim was to verify whether remote CCT could be conducted safely and consistently. Future studies should address this limitation by refining implementation methods, setting a larger sample size, and including structured qualitative interviews to gain a deeper understanding of participants’ experiences and perceptions.

Due to differences in measurement scales and participant characteristics, direct comparisons are difficult. However, the effectiveness of r-CCT was evaluated by assessing participants’ cognitive function, functional outcomes, and QOL before intervention, after intervention, and 3 months post r-CCT implementation to determine whether the training effects showed a trend similar to those of face-to-face CCT. The 4 cognitive domains targeted by r-CCT (ie, prospective memory, attention, learning and memory, and executive function) exhibited the following specific trends:

Prospective memory was measured using a belongings task; however, all 3 participants showed a decline in performance levels immediately after the intervention. By the 3-month follow-up, their performance had returned to preintervention levels, with no stable scores or improvement observed using r-CCT. Attention function was measured using the TMT-A, digit symbol, and digit span subtests, whereas executive function was measured using the TMT-B. In addition, 2 or 3 participants demonstrated an improvement in performance immediately after training, which was sustained at the 3-month follow-up. Learning and memory were measured using the JVLT and the story recall subtest. Furthermore, 2 to 3 participants demonstrated improved performance immediately after training; however, 2 participants exhibited a decline in performance at the 3-month follow-up. These results suggest that r-CCT may have beneficial effects on attention, learning and memory, and executive function, with prospective memory representing a potential exception. Furthermore, the 3-month follow-up results indicate that performance levels tend to remain stable, with the exception of learning and memory.

Previous studies on face-to-face CCT have found that, immediately after intervention, participants in the intervention group showed significant improvements across 4 cognitive-functional domains—prospective memory, attention, learning and memory, and executive function—relative to those receiving regular treatment [8,13]. This trend was observed in the 3 cognitive functions, excluding prospective memory, showing improvement trends similar to previous studies. However, the lack of improvement in prospective memory may be attributable to inconsistencies between the assessment tasks employed and the intervention content. The r-CCT uses a strategy called “the usual place,” which involves designating a specific location within the home—referred to as “home”—where essential items, such as keys and wallets, are consistently placed to prevent them from being forgotten [13]. This “usual place” strategy was particularly emphasized during training and was the only strategy to be included in all homework assignments. By contrast, in the belongings task, the examiner hides the participant’s belongings at the start of the cognitive function test and then asks them to recall the hidden items and their locations at the end of the test. A possible explanation for the lack of improvement in cognitive function following remote CCT is that the “usual place” strategy may have become integrated into participants’ daily routines, making it challenging for them to recall items hidden in locations outside of their homes during the belongings task. Future research on prospective memory should consider the careful selection of alternative assessment methods and inclusion of larger sample sizes.

Furthermore, previous studies on face-to-face CCT have reported that, at the 3-month follow-up, the intervention group showed significant improvements across 4 cognitive domains (prospective memory, attention, learning and memory, and executive function) compared with those receiving regular treatment [8,13]. This trend was observed in 3 cognitive functions excluding learning and memory, yielding results similar to those reported in previous studies. However, the lack of improvement in learning and memory in this study may be attributed to the nature of the homework assignments. Habit learning in individuals with schizophrenia is generally preserved, and declarative memory acquired through habitual practice is less likely to be forgotten [29]. CCT is designed to facilitate habit formation by assigning homework after each training session, enabling participants to apply strategies in their daily lives. However, since the training was conducted remotely this time, the implementation of homework was only confirmed with participants, and no visual confirmation was made. As a result, the extent to which participants actually implemented the homework remains unclear, which may explain why improvements in language memory were not sustained at the 3-month follow-up. Future studies should address these limitations by incorporating methods more suitable for remote settings. For example, measures such as having participants display the contents of their homework on the screen during homework confirmation or extending the time allocated for homework confirmation could be considered.

In this study on social functioning, SFS-J was used. However, when looking at the specific results, the scores of 2 participants improved immediately after the intervention; however, this improvement was not sustained at the 3-month follow-up. Scores on the JSQLS remained relatively stable in 2 out of 3 participants immediately after the intervention and in all 3 participants at the 3-month follow-up. In previous studies on face-to-face CCT targeting Japanese participants, effect sizes for functional indicators such as functional capacity and subjective QOL were reported to range from 0.47 to 1.56, suggesting that these effects were greater than those observed for cognitive function [14]. As this is a case study, effect sizes were not calculated, making direct comparisons with previous research difficult. However, the observed improvement trend in JSQLS scores immediately after the intervention and the sustained trend at the 3-month follow-up may reflect similar improvement trends observed in previous studies. However, the lack of stable SFS-J scores at the 3-month follow-up differs from the findings of previous studies. These findings may have been influenced by the participants’ living environment. Social life functioning is influenced by factors like motivation and social support [29]. Participants A, B, and C worked at the same social welfare facility, and the results may have been influenced by events at the facility and the nature of their work. Future studies may need to recruit participants from a wider population.

Overall, the results indicate that training without moving is relatively less burdensome for participants with schizophrenia as well as feasible to implement. This study has several limitations. First, the participants gathered at the same facility and received technical support during training; however, this format may not substantially reduce the burden on patients compared with traditional face-to-face interventions. Furthermore, it should be noted that devices such as iPads, television screens, and speakerphones may not be easily available or accessible to all participants and facilities. However, if the use of remote devices allows participants to receive training without having to visit the therapist’s location, then it should facilitate their participation and make it possible for more patients with schizophrenia to receive CCT. The authors suggest that if training could be developed in the future to allow therapists and participants to participate remotely with ease, then higher participation rates could be achieved. Although this was a small-scale pilot study, it identified specific challenges in implementing cognitive training using remote devices and provided initial insights into the practical feasibility of r-CCT. While several instances of unstable communication occurred during training sessions, no serious issues related to privacy or connectivity were reported, and no complaints were received from participants or assistants. In this study, the internet-based environment was set up smoothly by providing assistants with clear instructions about the entire process. If the operations and settings themselves were simplified slightly, participants may be able to operate the remote devices independently.

Without this study, it would be difficult to proceed with new research projects in this field. Currently, based on the results of this study, we are conducting a large-scale study with all participants who are able to participate from their homes. This new research is positioned as a separate project from the current research and is planned to be reported as an independent study in the future. Therefore, the data being collected currently are not included in this study.

### Limitations and Future Challenges

This study had some limitations. First, the small sample and the fact that participants were recruited from the same welfare facility made it difficult to generalize the results, which may have been strongly influenced by the facility that recruited the participants. Second, a target group was not established and a randomized controlled trial was not conducted; therefore, the effects of the intervention were not rigorously proven. However, this study was a preliminary study to explore the feasibility and acceptability of remote compensatory cognitive function improvement therapy. It was difficult to conduct a randomized controlled trial due to resource and practical constraints. While acknowledging that the absence of randomization and a control group may reduce internal validity and introduce selection bias, efforts were made to avoid arbitrary selection by researchers in the recruitment process. Participants were recruited from four social welfare facilities, with the cooperation and assistance of facility managers. In the initial approach, researchers did not directly approach participants but instead requested managers to approach them. By delegating the selection process to facility staff rather than researchers directly determining participants, the influence of researcher bias was minimized. Therefore, although this was not an intervention conducted under strict control, it reflected a natural implementation method tailored to the local situation and provided valuable insights for future implementation and research design. Third, as the subjects were Japanese, their schooling and life backgrounds differ slightly from subjects in previous studies; therefore, the results of this study should be interpreted with caution and replicated in future studies. Fourth, the intervention in this study was designed to be remote; however, it was not entirely home-based. Participants attended familiar welfare facilities and received technical support from assistants during. The assistants primarily provided support with device operation, while therapists conducted the intervention remotely. Although this partially remote intervention was an appropriate option for participants who were unfamiliar with digital devices, its ecological validity was limited due to the discrepancy between the participants’ actual living environments and the intervention setting. Such contextual factors are critically important when evaluating the feasibility of the intervention and should be systematically assessed alongside changes in cognitive function in future expansion studies.

This study also has strengths. First, although it was a case study, it found that cognitive function and QOL immediately after the r-CTT training were generally similar to those after face-to-face CCT, except for prospective memory. Second, the training’s effects on QOL were found to possibly persist after 3 months.

### Conclusion

This study examined the acceptability and feasibility of distance training for patients with schizophrenia participating in r-CCT. The study was conducted remotely to avoid participant movement, although internet-based problems were simultaneously considered. The 3 participants attended more than two-thirds of the 12 training sessions and expressed few negative comments about the remote training. In addition, cognitive functions targeted by r-CCT, except for prospective memory, could usually be maintained or improved immediately after training, even with r-CCT. However, whether this effect persists three months later should be thoroughly researched in the future. Meanwhile, it was suggested that patients could benefit in terms of QOL from the training, even when the remote method is used. These findings demonstrate that the remote method, depending on how it is used, may lead to improved training participation and participant acquisition. These are crucial findings regarding the acceptability and feasibility of r-CCT for patients with schizophrenia, and they indicate that the remote approach is likely to be suitable in Japan.

## Abbreviations

CCT: compensatory cognitive training
CRT: cognitive remediation therapy
JSQLS: Japanese Schizophrenia Quality of Life Scale
JVLT: Japanese Verbal Learning Test
QOL: quality of life
r-CCT: remote compensatory cognitive training
RBMT: Rivermead Behavioral Memory Test
SANS: Scale of Negative Symptoms
SAPS: Scale of Positive Symptoms
SFS-J: Social Functioning Scale Japanese version
TMT-A: Trail Making Test Part A
TMT-B: Trail Making Test Part B
WAIS-IV: Wechsler Adult Intelligence Scale–Fourth Edition
